# Potentially functional polymorphisms in the *LIN28B* gene contribute to neuroblastoma susceptibility in Chinese children

**DOI:** 10.1111/jcmm.12846

**Published:** 2016-03-29

**Authors:** Jing He, Tianyou Yang, Ruizhong Zhang, Jinhong Zhu, Fenghua Wang, Yan Zou, Huimin Xia

**Affiliations:** ^1^ Department of Pediatric SurgeryGuangzhou Women and Children's Medical CenterGuangzhou Medical UniversityGuangzhouGuangdongChina; ^2^ Sun Yat‐sen University Cancer CenterState Key Laboratory of Oncology in South ChinaDepartment of Experimental ResearchCollaborative Innovation Center for Cancer MedicineGuangzhouGuangdongChina; ^3^ Molecular Epidemiology Laboratory and Department of Laboratory MedicineHarbin Medical University Cancer HospitalHarbinHeilongjiangChina

**Keywords:** *LIN28B*, polymorphism, neuroblastoma, genetic susceptibility

## Abstract

Neuroblastoma is the most commonly diagnosed solid tumour outside the central nervous system in children. However, genetic factors underlying neuroblastoma remain largely unclear. Previous genome‐wide association study indicated that lin‐28 homolog B (LIN28B) might play an important role in the development of neuroblastoma and also contributed to its poor overall survival. With the purpose to evaluate the association between *LIN28B* gene polymorphisms and neuroblastoma susceptibility in Southern Chinese population, we conducted this study with 256 neuroblastoma cases and 531 cancer‐free controls. Four potentially functional polymorphisms (rs221634 A>T, rs221635 T>C, rs314276 C>A and rs9404590 T>G) were genotyped using Taqman method. Odds ratios (ORs) and 95% confidence intervals (CIs) were calculated to assess the associations between the selected single nucleotide polymorphisms (SNPs) and neuroblastoma susceptibility. We also performed genotype‐phenotype association analysis to explore the effects of the selected SNPs on *LIN28B* gene transcripts. Our results indicated that the rs221634 TT genotype was associated with an increased neuroblastoma risk (TT *versus* AA/AT: adjusted OR = 1.50, 95% CI = 1.04–2.17). The association was more pronounced in males, patients with tumour of mediastinum origin, as well as patients in early clinical stages. Moreover, overall analysis and stratified analysis also showed an increased risk of neuroblastoma for carrier of the 2–4 risk genotypes. In summary, these results indicated that the *LIN28B* rs221634 A>T polymorphism was associated with an increased neuroblastoma risk in Southern Chinese children. These findings need further validation in large studies with different ethnicities involved.

## Introduction

Neuroblastoma, derived from primitive sympathetic neural precursors, is the most commonly identified solid tumour outside the central nervous system in children 1, 2. The incidence rate of neuroblastoma in the live births in the United States is about 1/7000, with approximately 700 new cases per year 3. In contrast to adult tumours, neuroblastoma is quite rare, around 7.7 cases per million in China 4. Less than 40% of neuroblastoma patients can survival more than 5‐year after diagnosis 5. Survivors treated with multimodality therapy were more prone to develop chronic health conditions than those only treated with surgery. When compared to their siblings, survivors were less likely to been employed and get married, and attended to have low income 5. Therefore, neuroblastoma has become a great burden and challenge for their families and public health 6.

Epidemiology study searching for risk factors for neuroblastoma suggested that paternal exposures to wood dust, solders, electrical equipment and radiation sources, hydrocarbons (*e.g*., diesel fuel), lacquer thinner, and turpentine might associate with the increased incidence of neuroblastoma in offspring; however, the associations with most of the factors were not statistically significant 7, 8. Even in the presence of the paternal exposure to the same risk environmental factors, only a small portion of offspring of affected father developed neuroblastoma in the lifetime. Collectively, epidemiology studies failed to discovery common environmental exposures that can definitely influence neuroblastoma risk. On the other hand, accumulating evidence suggests that genetic factors also play essential roles in the development of neuroblastoma 9, 10, 11, 12. To date, genetic events that underlie the neuroblastoma remain largely unclear.

Understanding of the genetic basis is a key component of preventive oncology. The knowledge on human genome and rapid advances in genotyping technologies have made genome‐wide association studies (GWASs) possible for human diseases, including cancer 13. As so far, five neuroblastoma GWASs have been performed, and several neuroblastoma susceptibility genes have been identified such as *CASC15*/*CASC14* 14, *LMO1* 15, *HACE1* and lin‐28 homolog B (*LIN28B*) 16, *BARD1* limited to high‐risk neuroblastoma 17, as well as three low‐risk neuroblastoma susceptibility genes (*DUSP12* at 1q23.3, *DDX4* and *IL31RA* both at 5q11.2, and *HSD17B12* at 11p11.2) 18. In the expanded GWAS carried out by Diskin *et al*. 16 with a total of 2817 neuroblastoma cases and 7473 controls, two novel neuroblastoma susceptibility loci (*HACE1* rs4336470 C>T and *LIN28B* rs17065417 A>C) were identified. The same study also demonstrated that depletion of LIN28B protein might lead to significant growth inhibition *in vitro*, and high expression of LIN28B was associated with worse overall survival in primary neuroblastomas 16. Currently, the association established in this study was only evaluated in Italians 19 and Northern Chinese population 20. Given the biological importance of *LIN28B* and its implication in neuroblastomas, we performed the current hospital‐based case–control study to investigated the association between four potentially functional single nucleotide polymorphisms (SNPs) in the *LIN28B* gene and neuroblastoma susceptibility in Southern Chinese children.

## Materials and methods

### Patients and controls

All of the neuroblastoma cases received treatments from the Guangzhou Women and Children's Medical Center as described previously 21. Briefly, we recruited 256 genetically unrelated ethnic Han Chinese children with newly diagnosed and histopathologically confirmed primary neuroblastoma mainly between February 2010 and November 2015. We also included a total of 531 age‐, gender‐, race‐matched cancer‐free volunteers recruited from the same hospital 21. The response rate was approximately 90% and 95% for neuroblastoma subjects and cancer‐free controls, respectively. The study was approved by the Institutional Review Board of Guangzhou Women and Children's Medical Center. All of the participants provided written informed consent by themselves or their guardians.

### SNP selection and genotyping

We chose potentially functional SNPs of interest from dbSNP database (http://www.ncbi.nlm.nih.gov/) and an online tool, SNPinfo (http://snpinfo.niehs.nih.gov/) based on the following criteria: (*i*) minor allele frequency >5% for Chinese Han subjects; (*ii*) potentially functional, such as affecting the binding capacity of transcription factor binding sites or microRNA binding sites, or leading to amino acids alterations. Four potentially functional SNPs (rs221634 A>T, rs221635 T>C, rs314276 C>A, and rs9404590 T>G) in the *LIN28B* gene that met these criteria were chosen in this study. The detailed information of these four SNPs as well as the previous GWAS‐identified rs17065417 A>C were described in Table S1. An additional of 40 SNPs, in linkage disequilibrium (*R* ^2^ > 0.8) with these four SNPs, were captured by using SNPinfo (http://snpinfo.niehs.nih.gov/). SNP genotyping was performed by Taqman real‐time PCR system as reported elsewhere 22. To validate the accuracy of genotyping results from Taqman real‐time PCR and for quality control, approximately 10% of the samples were randomly selected and genotyped with sequencing. The concordance for the quality control samples was 100%.

### Statistical analysis

The chi‐squared test was used to compared neuroblastoma cases and controls, regarding demographic variables. Test for Hardy–Weinberg equilibrium (HWE) in control subjects was performed for each SNP by a goodness‐of‐fit chi‐squared test. The associations between the selected SNPs and neuroblastoma susceptibility were determined by computing the odds ratios (ORs) and 95% confidence intervals (CIs), using unconditional logistic regression analyses. Stratified analysis was performed for subgroups by age, gender, tumour sites, and clinical stages. We also conducted genotype‐based mRNA expression analysis as described previously 22, 23, 24. All statistical analyses were performed with SAS software (Version 9.1; SAS Institute, Cary, NC, USA). *P*‐values less than 0.05 were considered as statistically significant.

## Results

### General characteristics of the subjects

As shown in Table S2, we enrolled 256 neuroblastoma patients with an average age of 30.87 ± 26.45 months and 531 cancer‐free subjects with an average age of 29.73 ± 24.86 months. There was no significant difference between the case and control groups, regarding age (*P* = 0.239) and gender (*P* = 0.333). According to the INSS criteria 2, there were 54 (21.09%), 65 (25.39%), 44 (17.19%), 77 (30.08%), and 9 (3.52%) individuals with clinical stage I, II, III, IV, and 4s neuroblastoma, respectively. In term of the tumour sites, 46 (17.97%), 87 (33.98%), and 90 (35.16%) neuroblastomas occurred in adrenal gland, retroperitoneal region, and mediastinum, respectively.

### Associations of *LIN28B* gene SNPs with neuroblastoma susceptibility

Of the included subjects, 247 cases and 530 controls were successfully genotyped. The genotype frequencies of all the four selected SNPs in controls were in HWE (*P* = 0.228 for rs221634 A>T, *P* = 0.527 for rs221635 T>C, *P* = 0.756 for rs314276 C>A, and *P* = 0.786 for rs9404590 T>G). The genotype frequencies of the four SNPs in neuroblastoma cases and cancer‐free controls were shown in Table 1. Our results indicated that carriers of the rs221634 TT genotype had significantly increased neuroblastoma risk at an adjusted OR of 1.50 (95% CI = 1.04–2.17) when compared with carriers of the AA/AT genotypes. We also found that the rs221634 C allele was borderline associated with decreased neuroblastoma risk (TC *versus* TT: adjusted OR = 0.74, 95% CI = 0.52–1.04, *P* = 0.081; CC/TC *versus* TT: adjusted OR = 0.74, 95% CI = 0.54–1.03, *P* = 0.078). Moreover, no significant association or any trend was observed for the rest two polymorphisms. While the risk genotypes were combined, we found a significant trend towards an increased neuroblastoma risk with more risk genotypes (adjusted OR = 1.21, 95% CI = 1.02–1.43, *P* = 0.026). We also observed that subjects carrying 2–4 risk genotypes had a significantly increased neuroblastoma risk (adjusted OR = 1.53, 95% CI = 1.12–2.08, *P* = 0.007) when compared with those carrying 0–1 risk genotypes.

**Table 1:** Genotype frequencies of *LIN28B* gene polymorphisms and neuroblastoma susceptibility

Genotype	Cases (*N* = 247)	Controls (*N* = 530)	*P* *	Crude OR (95% CI)	*P*	Adjusted OR (95% CI)†	*P* †
rs221634 (HWE = 0.228)							
AA	74 (29.96)	163 (30.75)		1.00		1.00	
AT	113 (45.75)	274 (51.70)		0.91 (0.64–1.29)	0.592	0.92 (0.64–1.30)	0.621
TT	60 (24.29)	93 (17.55)		1.42 (0.93–2.17)	0.105	1.42 (0.93–2.18)	0.105
Additive			0.077	1.17 (0.94–1.45)	0.163	1.17 (0.94–1.45)	0.160
Dominant	173 (70.04)	367 (69.25)	0.823	1.04 (0.75–1.44)	0.823	1.04 (0.75–1.45)	0.798
Recessive	187 (75.41)	437 (82.45)	0.028	**1.51 (1.05–2.18)**	**0.028**	**1.50 (1.04–2.17)**	**0.030**
rs221635 (HWE = 0.527)							
TT	176 (71.26)	345 (65.09)		1.00		1.00	
TC	64 (25.91)	168 (31.70)		0.75 (0.53–1.05)	0.093	0.74 (0.52–1.04)	0.081
CC	7 (2.83)	17 (3.21)		0.81 (0.33–1.98)	0.640	0.80 (0.33–1.97)	0.630
Additive			0.232	0.79 (0.60–1.06)	0.118	0.79 (0.59–1.05)	0.105
Dominant	71 (28.74)	185 (34.91)	0.089	0.75 (0.54–1.05)	0.089	0.74 (0.54–1.03)	0.078
Recessive	240 (97.17)	513 (96.79)	0.779	0.88 (0.36–2.15)	0.779	0.88 (0.36–2.14)	0.771
rs314276 (HWE = 0.756)							
CC	125 (50.61)	254 (47.92)		1.00		1.00	
CA	96 (38.87)	228 (43.02)		0.86 (0.62–1.18)	0.340	0.86 (0.62–1.18)	0.346
AA	26 (10.53)	48 (9.06)		1.10 (0.65–1.86)	0.719	1.11 (0.66–1.87)	0.702
Additive			0.515	0.97 (0.77–1.23)	0.810	0.98 (0.77–1.23)	0.827
Dominant	122 (49.39)	276 (52.08)	0.486	0.90 (0.66–1.22)	0.486	0.90 (0.67–1.22)	0.497
Recessive	221 (89.47)	482 (90.94)	0.516	1.18 (0.71–1.95)	0.516	1.19 (0.72–1.97)	0.503
rs9404590 (HWE = 0.786)							
TT	130 (52.63)	286 (53.96)		1.00		1.00	
TG	100 (40.49)	205 (38.68)		1.07 (0.78–1.47)	0.662	1.07 (0.78–1.47)	0.658
GG	17 (6.88)	39 (7.36)		0.96 (0.52–1.76)	0.892	0.96 (0.52–1.77)	0.901
Additive			0.883	1.02 (0.80–1.30)	0.859	1.02 (0.80–1.30)	0.851
Dominant	117 (47.37)	244 (46.04)	0.471	1.06 (0.78–1.43)	0.729	1.06 (0.78–1.43)	0.723
Recessive	230 (93.12)	491 (92.64)	0.811	0.93 (0.52–1.68)	0.811	0.93 (0.52–1.69)	0.819
Combined effect of risk genotypes							
0	45 (18.22)	122 (23.02)		1.00		1.00	
1	48 (19.43)	132 (24.91)		0.99 (0.61–1.59)	0.953	1.00 (0.62–1.60)	0.984
2	132 (53.44)	230 (43.40)		**1.56 (1.04–2.33)**	**0.032**	**1.56 (1.04–2.34)**	**0.030**
3	21 (8.50)	46 (8.68)		1.24 (0.67–2.30)	0.500	1.26 (0.68–2.34)	0.471
4	1 (0.40)	0 (0.00)		/	/	/	/
Trend			0.044	**1.21 (1.02–1.42)**	**0.028**	**1.21 (1.02–1.43)**	**0.026**
0–1	93 (37.65)	254 (47.92)		1.00		1.00	
2–4	154 (62.35)	276 (52.08)	0.007	**1.52 (1.12–2.08)**	**0.008**	**1.53 (1.12–2.08)**	**0.007**

The significant results were in bold, if the 95% CI excluded 1 or *P* < 0.05. *Chi‐squared test for genotype distributions between neuroblastoma patients and controls. ^†^Adjusted for age and gender.

### Stratified analysis

We next analyed the association of the rs221634 A>T and combined risk genotypes with neuroblastoma risk by stratification analysis (Table 2). We found that the increased risk associated with the rs221634 TT variant genotype was more pronounced in males (adjusted OR = 2.11, 95% CI = 1.32–3.40), patients with tumour of mediastinum origin (adjusted OR = 1.99, 95% CI = 1.19–3.33) as well as patients in early clinical stages (adjusted OR = 1.62, 95% CI = 1.01–2.58). As to the risk genotypes, the significant association between the 2–4 risk genotypes and neuroblastoma risk was statistically significant in younger infants (adjusted OR = 1.83, 95% CI = 1.12–2.99), males (adjusted OR = 1.65, 95% CI = 1.10–2.47), patients with tumour originated from retroperitoneal (adjusted OR = 1.68, 95% CI = 1.03–2.73) and mediastinum (adjusted OR = 1.70, 95% CI = 1.06–2.72), as well as patients in clinical stages III/IV (adjusted OR = 1.61, 95% CI = 1.06–2.46).

**Table 2:** Stratification analysis of risk genotypes with neuroblastoma susceptibility

Variables	rs221634 (cases/controls)		OR (95% CI)	*P*	Adjusted OR* (95% CI)	*P* *	Combined		OR (95% CI)	*P*	Adjusted OR* (95% CI)	*P* *
	AA/AT	TT					0–1	2–4				
Age, month												
≤18	74/192	23/41	1.46 (0.82–2.59)	0.202	1.44 (0.81–2.57)	0.212	33/113	64/120	**1.83 (1.12–2.99)**	**0.017**	**1.83 (1.12–2.99)**	**0.016**
>18	113/245	37/52	1.54 (0.96–2.49)	0.075	1.55 (0.96–2.50)	0.072	60/141	90/156	1.36 (0.91–2.02)	0.134	1.36 (0.91–2.02)	0.135
Gender												
Females	81/186	18/46	0.90 (0.49–1.64)	0.729	0.89 (0.49–1.63)	0.712	39/109	60/123	1.36 (0.85–2.20)	0.204	1.37 (0.85–2.21)	0.201
Males	106/251	42/47	**2.12 (1.32–3.40)**	**0.002**	**2.11 (1.32–3.40)**	**0.002**	54/145	94/153	**1.65 (1.10–2.47)**	**0.015**	**1.65 (1.10–2.47)**	**0.015**
Sites of origin												
Adrenal gland	37/437	9/93	1.14 (0.53–2.45)	0.731	1.12 (0.52–2.42)	0.770	21/254	25/276	1.10 (0.60–2.01)	0.767	1.09 (0.59–2.00)	0.783
Retroperitoneal	63/437	18/93	1.34 (0.76–2.37)	0.311	1.37 (0.77–2.42)	0.284	29/254	52/276	**1.65 (1.02–2.68)**	**0.043**	**1.68 (1.03–2.73)**	**0.038**
Mediastinum	62/437	26/93	**1.97 (1.18–3.28)**	**0.009**	**1.99 (1.19–3.33)**	**0.008**	31/254	57/276	**1.69 (1.06–2.71)**	**0.028**	**1.70 (1.06–2.72)**	**0.027**
Others	18/437	6/93	1.57 (0.61–4.06)	0.355	1.64 (0.63–4.26)	0.311	8/254	16/276	1.84 (0.77–4.37)	0.167	1.87 (0.78–4.44)	0.159
Clinical stages												
I+II+4s	92/437	31/93	1.58 (1.00–2.52)	0.053	**1.62 (1.01–2.58)**	**0.044**	48/254	75/276	1.44 (0.96–2.15)	0.075	1.44 (0.97–2.15)	0.074
III+IV	89/437	26/93	1.37 (0.84–2.24)	0.206	1.34 (0.82–2.21)	0.242	42/254	73/276	**1.60 (1.06–2.43)**	**0.027**	**1.61 (1.06–2.46)**	**0.025**

The significant results were in bold, if the 95% CI excluded 1 or *P* < 0.05. *Adjusted for age and gender.

### Genotype‐based mRNA expression analysis

We performed genotype‐based mRNA expression for the rs221634 A>T and rs221635 T>C polymorphisms. However, no significant alteration in mRNA expression level was observed in any ethnicity or the whole population (Table 3).

**Table 3:** *LIN28B* mRNA expression by the genotypes of rs221634 A>T and rs221635 T>C, using data from the HapMap*

Population	rs221634 A>T					rs221635 T>C				
	Genotypes	No.	Mean ± S.D.	*P* †	*P*_trend_ ‡	Genotypes	No.	Mean ± S.D.	*P* †	*P*_trend_ ‡
CEU§	AA	44	5.93 ± 0.07		0.538	TT	77	5.92 ± 0.06		0.495
	AT	38	5.92 ± 0.07	0.459		TC	12	5.90 ± 0.07	0.292	
	TT	7	5.90 ± 0.05	0.315		CC	1	5.89	0.551	
	Dominant	45	5.91 ± 0.07	0.338		Dominant	13	5.90 ± 0.07	0.245	
	Recessive	82	5.92 ± 0.07	0.410		Recessive	89	5.92 ± 0.07	0.589	
YRI	AA	7	5.95 ± 0.04		0.449	TT	29	5.93 ± 0.06		0.435
	AT	40	5.93 ± 0.07	0.321		TC	46	5.93 ± 0.06	0.644	
	TT	43	5.92 ± 0.06	0.171		CC	15	5.92 ± 0.07	0.218	
	Dominant	83	5.92 ± 0.06	0.232		Dominant	61	5.92 ± 0.06	0.423	
	Recessive	47	5.93 ± 0.06	0.460		Recessive	75	5.93 ± 0.06	0.225	
Asian§	AA	22	5.93 ± 0.05		0.171	TT	56	5.94 ± 0.06		0.588
	AT	46	5.95 ± 0.06	0.221		TC	29	5.94 ± 0.05	0.728	
	TT	21	5.92 ± 0.05	0.639		CC	5	5.92 ± 0.03	0.325	
	Dominant	67	5.94 ± 0.06	0.471		Dominant	34	5.94 ± 0.05	0.523	
	Recessive	68	5.94 ± 0.06	0.158		Recessive	85	5.94 ± 0.06	0.332	
All§	AA	73	5.93 ± 0.06		0.348	TT	162	5.93 ± 0.06		0.229
	AT	124	5.93 ± 0.07	0.953		TC	87	5.93 ± 0.06	0.506	
	TT	71	5.92 ± 0.06	0.217		CC	21	5.91 ± 0.06	0.096	
	Dominant	195	5.93 ± 0.06	0.634		Dominant	108	5.92 ± 0.06	0.236	
	Recessive	197	5.93 ± 0.06	0.146		Recessive	249	5.93 ± 0.06	0.113	

*Genotyping data and mRNA expression levels for *LIN28B* by genotypes were obtained from the HapMap phase II release 23 data from EBV‐transformed lymphoblastoid cell lines from 270 individuals. ^†^Two‐side Student's *t*‐test within the stratum. ^‡^ *P*‐values for the trend test of *LIN28B* mRNA expression among three genotypes for each SNP from a general linear model. ^§^There were missing data because genotyping data were not available.

## Discussion

In the current hospital‐based case–control study, we found the *LIN28B* gene rs221634 A>T polymorphism was associated with neuroblastoma susceptibility in a Southern Chinese population. To the best of our knowledge, the present investigation was the first one to verify the significant association between rs221634 TT genotype and an increased neuroblastoma risk in Chinese children under the recessive model.

The *LIN28B* gene is located on chromosome 6q21, consisting of seven exons. Its protein product belongs to the lin‐28 family, which are featured by a cold‐shock domain and a pair of CCHC zinc finger domains 25. The *LIN28B* gene was first identified by Guo *et al*. 26 in human hepatocellular carcinoma (HCC). They found overexpression of LIN28B in most HCC cell lines as well as HCC tissue samples. The LIN28B protein shows cell cycle‐dependent nuclear translocation, and its overexpression can promote cancer cell proliferation. They also found the 3′UTR of the *LIN28B* gene contains complementary sites to let‐7 microRNA. *LIN28B* gene may be negatively regulated by let‐7, which consequentially facilitates cellular transformation 26. Interestingly, later studies indicated that *LIN28B* rs314276 C>A polymorphism, which is included in our current study, is associated with timing of puberty 27, growth in height from birth to adulthood 28, finger‐length ratio 29, growth in height from birth to adulthood 28, childhood obesity and age at menarche 30, and central precocious puberty and early puberty in girls 31. Moreover, this variant was also found to be associated with the risk and survival of cancer, such as epithelial ovarian cancer 32, colon cancer 33, 34, non‐small cell lung cancer 35, oral cavity cancer 36, and neuroblastoma 16. LIN28B protein was found to be unregulated in colon tumours, and the overexpression correlated with reduced patient survival and increased probability of tumour recurrence 34, 37; besides, LIN28B can promote migration, invasion, and malignant transformation of cells 38. Furthermore, studies also found that the overexpression of LIN28B was associated with poor prognosis in head and neck cancer 39, oral squamous cell carcinoma patients 40, 41, gastric cancer 42, breast cancer 43, ovarian cancer 44, and neuroblastoma 16, 45, 46.

As so far, three GWASs have been performed to identify the neuroblastoma susceptibility loci 14, 15, 16, as well as susceptibility loci for high‐risk neuroblastoma 17 and low‐risk neuroblastoma 18. In the first GWAS with a total of 1752 neuroblastoma cases and 4171 controls, three genetic variants (rs6939340 A>G, rs4712653 T>C and rs9295536 C>A) at chromosome 6p22 were identified to be associated with neuroblastoma susceptibility 14. In the expanded GWAS with 2251 neuroblastoma cases and 6097 controls restricted to European ancestry, four polymorphisms (rs110419 A>G, rs4758051G>A, rs10840002 A>G and rs204938 A>G) in the *LMO1* locus on 11p15.4 were found to confer neuroblastoma susceptibility 15. In the latest expanded GWAS, a total of 2101 neuroblastoma cases and 4202 controls were included in the discovery stage, while 351 cases and 780 controls from Italy, as well as 365 cases and 2491 controls from African‐Americans were recruited to validate the significant variants. Authors found that five polymorphisms at the *HACE1* gene and the *LIN28B* rs17065417 A>C polymorphism were associated with neuroblastoma susceptibility 16. In a validation study with a total of 370 cases and 809 controls in an Italian population, Capasso *et al*. 19 tested cumulative effects of neuroblastoma risk variants using genotype data of rs17065417 A>C polymorphism from the validation stage 16, which was already demonstrated to associate with neuroblastoma susceptibility. In the Chinese subjects, Lu *et al*. 20 performed a hospital‐based case–control study with 244 neuroblastoma cases and 305 controls. They were not able to repeat any reported association between selected polymorphisms in the *LIN28B* gene and neuroblastoma susceptibility. According the dbSNP (http://www.ncbi.nlm.nih.gov/SNP), there are at least 6640 SNPs identified in the *LIN28B* gene. Considering the important role of *LIN28B* in carcinogenesis, we chose four potentially functional SNPs to investigate the association of genetic variants in *LIN28B* with neuroblastoma susceptibility. The GWAS‐identified *LIN28B* rs17065417 A>C polymorphism was also genotyped, although online tool SNPinfo predicated that only AA wild type of this SNP was present in Asians, including Chinese. As expected, we only identify AA genotype in Southern Chinese children. Of the four investigated polymorphisms with all three types of genotypes identified in the study population, we found that only the *LIN28B* rs221634 TT genotype was associated with an increased neuroblastoma risk. We also found the subjects with 2–4 risk genotypes had a significantly increased neuroblastoma risk. Failure to find the association for the other three polymorphisms in this study may be ascribe to the limited sample size as well as the weak effects of these SNPs. Overall, our findings provided further evidence for the important role of *LIN28B* gene in the tumourigenesis of neuroblastoma.

Although we found the correlation between *LIN28B* gene rs221634 A>T polymorphism and neuroblastoma risk for the first time, several possible limitations in this study should be addressed. Firstly, because of the natural of retrospective study design, selection and information bias might be unavoidable. These biases could only be reduced by frequency‐matching of cases and controls by age and sex, to some extent, since information on paternal exposures, dietary intake, and living environment was not available. Secondly, we only include four potentially functional SNPs in this study. Because of the absence of variant allele (<0.05) in Chinese, the previous GWAS‐identified rs17065417 A>C was not included in our final analysis. Thirdly, though this was the largest study in Chinese children, the sample size was relatively small. Only 256 neuroblastoma patients and 531 cancer‐free controls were included, which may lead to limited statistical power. Finally, rs221634 resides within a miRNA binding site in the 3′UTR of *LIN28B* gene. As predicted, three miRNAs (hsa‐miR‐548b‐5p, hsa‐miR‐548c‐5p and hsa‐miR‐548d‐5p) might bind to this miRNA binding site. Since genotype‐based mRNA expression analysis failed to show the genotype‐related alteration in *LIN28B* transcripts, we did not perform functional analysis for these SNPs.

In summary, in this study, we found that the *LIN28B* gene rs221634 A>T polymorphism might be associated with increased neuroblastoma risk in Southern Chinese childhood, especially for males, patients with tumour of mediastinum region, as well as patients in early clinical stages. However, well‐designed prospective studies are encouraged to verify our findings, with larger sample size and detailed information such as paternal exposures.

## Conflicts of interest

The authors confirm that there are no conflicts of interest.

## Supporting information


**Table S1** SNPs captured by the selected four LIN28B potentially functional SNPs and the GWAS identified rs17065417 A>C polymorphism as predicted by SNPinfo (http://snpinfo.niehs.nih.gov/) software.
**Table S2** Frequency distribution of demographic characteristics in neuroblastoma patients and controls.
