# Supplementary material for: Potentially functional polymorphisms in the LIN28B gene contribute to neuroblastoma susceptibility in Chinese children
Source: J Cell Mol Med. 2016 Mar 29;20(8):1534–41. doi: 10.1111/jcmm.12846 (PMC4956938; doi:10.1111/jcmm.12846)
Supplement: Supplementary file 1 — Table S1 SNPs captured by the selected four LIN28B potentially functional SNPs and the GWAS identified rs17065417 A>C polymorphism as predicted by SNPinfo (http://snpinfo.niehs.nih.gov/) software. Table S2 Frequency distribution of demographic characteristics in neuroblastoma patients and controls. [file JCMM-20-1534-s001.doc]

| **Supplemental Table 1.** SNPs captured by the selected four *LIN28B* potentially functional SNPs and the GWAS identified rs17065417 A>C polymorphism as predicted by SNPinfo (<http://snpinfo.niehs.nih.gov/>) software. | | | | | | | | | | | | | | | |
| --- | --- | --- | --- | --- | --- | --- | --- | --- | --- | --- | --- | --- | --- | --- | --- |
| **rs** | **Chr** | **Allele** | **LDsnp** | **Pop/R^2^** | **TFBS** | **miRNA (miRanda)** | **nsSNP** | **Polyphen** | **Reg**  **Potential** | **Conservation^a^** | **Nearby Gene** | **Distance (bp)** | **Allele** | **Asian** | **CHB** |
| **rs17065417** | **6** | **A/C** | **rs17065417** | **1** | **Y** | **--** | **--** | **--** | **0** | **0.006** | **LIN28B** | **1351||124932** | **A** | **1** | **1** |
| rs160600 | 6 | G/A | rs221634 | CHB/0.909 | -- | -- | -- | -- | NA | 0.031 | LIN28B | 86340||39943 | A | -- | 0.542 |
| rs221612 | 6 | G/C | rs221634 | CHB/1.000 | -- | -- | -- | -- | NA | NA | LIN28B | 92095||34188 | C | 0.491 | 0.5 |
| **rs221634** | **6** | **A/T** | **rs221634** | **1** | **--** | **Y** | **--** | **--** | **0** | **0.211** | **LIN28B** | **123165||3118** | **A** | **0.511** | **0.511** |
| rs160595 | 6 | G/A | rs221635 | CHB/1.000 | -- | -- | -- | -- | 0 | 0.009 | LIN28B | 88132||38151 | G | 0.811 | 0.804 |
| rs160598 | 6 | G/A | rs221635 | CHB/1.000 | -- | -- | -- | -- | 0 | 0.012 | LIN28B | 86935||39348 | G | -- | 0.804 |
| rs221626 | 6 | A/G | rs221635 | CHB/0.929 | -- | -- | -- | -- | 0 | 0 | LIN28B | 101319||24964 | A | 0.796 | 0.792 |
| rs221628 | 6 | A/G | rs221635 | CHB/1.000 | -- | -- | -- | -- | 0 | 0.005 | LIN28B | 107256||19027 | G | 0.794 | 0.801 |
| rs221629 | 6 | C/G | rs221635 | CHB/0.933 | -- | -- | -- | -- | 0.127304 | 0.07 | LIN28B | 108448||17835 | C | 0.787 | 0.789 |
| rs221633 | 6 | C/G | rs221635 | CHB/1.000 | -- | -- | -- | -- | 0 | 0.018 | LIN28B | 114271||12012 | C | 0.781 | 0.8 |
| **rs221635** | **6** | **C/T** | **rs221635** | **1** | **--** | **Y** | **--** | **--** | **0.006678** | **0.001** | **LIN28B** | **124751||1532** | **T** | **0.803** | **0.804** |
| rs221636 | 6 | A/T | rs221635 | CHB/1.000 | -- | Y | -- | -- | 0.041471 | 0.008 | LIN28B | 125041||1242 | A | 0.875 | 0.804 |
| rs364758 | 6 | A/C | rs221635 | CHB/0.963 | -- | -- | -- | -- | NA | 0 | LIN28B | 97733||28550 | A | 0.875 | 0.798 |
| rs379908 | 6 | C/T | rs221635 | CHB/0.963 | -- | -- | -- | -- | 0.002555 | 0.958 | LIN28B | 118756||7527 | T | 0.796 | 0.798 |
| rs364663 | 6 | T/A | rs314276 | CHB/0.867 | -- | -- | -- | -- | NA | 0.022 | LIN28B | 38266||88017 | A | 0.747 | 0.789 |
| rs4946651 | 6 | A/G | rs314276 | CHB/0.933 | -- | -- | -- | -- | 0.027264 | 0.003 | HACE1||RP3-439I14.1 | -61716||-14659 | A | 0.294 | 0.2 |
| rs11156429 | 6 | G/T | rs314276|rs9404590 | CHB/1.000|CHB/0.869 | -- | -- | -- | -- | NA | 0 | HACE1||RP3-439I14.1 | -56627||-19748 | G | 0.753 | 0.789 |
| rs12200251 | 6 | A/G | rs314276|rs9404590 | CHB/1.000|CHB/0.875 | -- | -- | -- | -- | 0.068222 | 0 | HACE1||RP3-439I14.1 | -74621||-1754 | G | -- | 0.244 |
| rs1322417 | 6 | A/G | rs314276|rs9404590 | CHB/0.841|CHB/0.966 | -- | -- | -- | -- | NA | 0 | LIN28B | 42272||84011 | G | -- | 0.774 |
| rs1475120 | 6 | G/A | rs314276|rs9404590 | CHB/1.000|CHB/0.875 | -- | -- | -- | -- | 0.175198 | 0 | RP3-439I14.1 | 5784||16249 | A | -- | 0.756 |
| rs167539 | 6 | C/A | rs314276|rs9404590 | CHB/1.000|CHB/0.875 | Y | -- | -- | -- | 0 | 0 | LIN28B | 5125||121158 | A | 0.734 | 0.756 |
| rs1744206 | 6 | C/G | rs314276|rs9404590 | CHB/1.000|CHB/0.869 | -- | -- | -- | -- | 0 | 0.042 | LIN28B | 19008||107275 | G | 0.263 | 0.211 |
| rs2095812 | 6 | C/G | rs314276|rs9404590 | CHB/1.000|CHB/0.869 | -- | -- | -- | -- | 0 | 0.002 | HACE1||RP3-439I14.1 | -76184||-191 | C | 0.747 | 0.789 |
| rs314262 | 6 | G/A | rs314276|rs9404590 | CHB/1.000|CHB/0.875 | -- | -- | -- | -- | 0 | 0.001 | RP3-439I14.1 | 10452||11581 | G | 0.244 | 0.244 |
| rs314263 | 6 | C/T | rs314276|rs9404590 | CHB/1.000|CHB/0.875 | -- | -- | -- | -- | 0.208481 | 1 | RP3-439I14.1 | 8576||13457 | T | 0.753 | 0.756 |
| rs314266 | 6 | C/T | rs314276|rs9404590 | CHB/1.000|CHB/0.875 | -- | -- | -- | -- | 0.019382 | 0 | LIN28B | 16394||109889 | T | 0.758 | 0.756 |
| rs314268 | 6 | G/A | rs314276|rs9404590 | CHB/0.967|CHB/0.902 | -- | -- | -- | -- | 0.108969 | 0.858 | LIN28B | 13055||113228 | A | 0.756 | 0.765 |
| rs314270 | 6 | T/C | rs314276|rs9404590 | CHB/1.000|CHB/0.869 | -- | -- | -- | -- | NA | 0 | LIN28B | 57953||68330 | C | 0.756 | 0.789 |
| rs314272 | 6 | G/A | rs314276|rs9404590 | CHB/0.968|CHB/0.847 | -- | -- | -- | -- | NA | 0 | LIN28B | 57081||69202 | G | 0.333 | 0.25 |
| rs314273 | 6 | T/G | rs314276|rs9404590 | CHB/1.000|CHB/0.869 | -- | -- | -- | -- | NA | 0 | LIN28B | 56959||69324 | G | 0.796 | 0.789 |
| rs314274 | 6 | A/C | rs314276|rs9404590 | CHB/1.000|CHB/0.875 | -- | -- | -- | -- | 0 | 0.003 | LIN28B | 8009||118274 | C | 0.735 | 0.756 |
| **rs314276** | **6** | **A/C** | **rs314276|rs9404590** | **1|CHB/0.874** | **Y** | **--** | **--** | **--** | **0** | **0** | **LIN28B** | **3076||123207** | **C** | **0.756** | **0.75** |
| rs314280 | 6 | G/A | rs314276|rs9404590 | CHB/1.000|CHB/0.875 | Y | -- | -- | -- | 0.098309 | 0.001 | RP3-439I14.1 | 16668||5365 | A | 0.244 | 0.244 |
| rs314286 | 6 | T/C | rs314276|rs9404590 | CHB/0.968|CHB/0.847 | -- | -- | -- | -- | NA | 0 | LIN28B | 31130||95153 | T | -- | 0.75 |
| rs314289 | 6 | T/C | rs314276|rs9404590 | CHB/1.000|CHB/0.875 | -- | -- | -- | -- | NA | 0 | LIN28B | 26011||100272 | C | 0.25 | 0.244 |
| rs314290 | 6 | A/G | rs314276|rs9404590 | CHB/1.000|CHB/0.909 | -- | -- | -- | -- | 0 | 0.035 | LIN28B | 22071||104212 | G | 0.769 | 0.838 |
| rs314291 | 6 | C/T | rs314276|rs9404590 | CHB/1.000|CHB/0.875 | -- | -- | -- | -- | NA | 0.004 | LIN28B | 19978||106305 | T | 0.744 | 0.756 |
| rs395962 | 6 | G/T | rs314276|rs9404590 | CHB/1.000|CHB/0.875 | -- | -- | -- | -- | 0.117108 | 0 | RP3-439I14.1 | 13249||8784 | G | 0.756 | 0.756 |
| rs454568 | 6 | C/T | rs314276|rs9404590 | CHB/0.968|CHB/0.847 | -- | -- | -- | -- | 0 | 0 | LIN28B | 37714||88569 | T | -- | 0.75 |
| rs457286 | 6 | C/A | rs314276|rs9404590 | CHB/0.968|CHB/0.847 | -- | -- | -- | -- | NA | 0.474 | LIN28B | 61288||64995 | C | 0.667 | 0.75 |
| rs7759938 | 6 | C/T | rs314276|rs9404590 | CHB/1.000|CHB/0.875 | -- | -- | -- | -- | NA | 0 | HACE1||RP3-439I14.1 | -71160||-5215 | T | 0.747 | 0.756 |
| rs9377684 | 6 | A/G | rs314276|rs9404590 | CHB/0.864|CHB/0.965 | -- | -- | -- | -- | 0 | 0.004 | LIN28B | 40441||85842 | A | 0.822 | 0.778 |
| rs9391253 | 6 | A/T | rs314276|rs9404590 | CHB/1.000|CHB/0.869 | -- | -- | -- | -- | 0 | 0 | HACE1||RP3-439I14.1 | -59822||-16553 | A | 0.756 | 0.789 |
| rs9404592 | 6 | A/G | rs314276|rs9404590 | CHB/0.913|CHB/1.000 | -- | -- | -- | -- | NA | 0 | LIN28B | 56962||69321 | G | 0.836 | 0.829 |
| **rs9404590** | **6** | **G/T** | **rs9404590|rs314276** | **1|CHB/0.874** | **--** | **--** | **Y** | **benign** | **0.295608** | **0.001** | **RP3-439I14.1** | **16844||5189** | **T** | **0.14** | **0.78** |
| SNP, single nucleotide polymorphism; GWAS, genome-wide association study; TFBS, transcription factor binding sites; LD, linkage disequilibrium; CHB, Han Chinese in Beijing, China  ^a^ Conservation score (17 Species) downloaded from UCSC genome bioinformatics web site (<http://genome.ucsc.edu/>) | | | | | | | | | | | | | | | |

| **Supplemental Table 2.** Frequency distribution of demographic characteristics in neuroblastoma patients and controls | | | | | |
| --- | --- | --- | --- | --- | --- |
| Variables | Cases (n=256) | | Controls (n=531) | | *P^a^* |
|  | No. | % | No. | % |  |
| Age range, month | 0-156 | | 0.07-156 | | 0.239 |
| Mean ± SD | 30.87 ± 26.45 | | 29.73 ± 24.86 | |  |
| ≤18 | 101 | 39.45 | 233 | 43.88 |  |
| >18 | 155 | 60.55 | 298 | 56.12 |  |
| Gender |  |  |  |  | 0.333 |
| Female | 103 | 40.23 | 233 | 43.88 |  |
| Male | 153 | 59.77 | 298 | 56.12 |  |
| Clinical stages |  |  |  |  |  |
| I | 54 | 21.09 |  |  |  |
| II | 65 | 25.39 |  |  |  |
| III | 44 | 17.19 |  |  |  |
| IV | 77 | 30.08 |  |  |  |
| 4s | 9 | 3.52 |  |  |  |
| NA | 7 | 2.73 |  |  |  |
| Sites of origin |  |  |  |  |  |
| Adrenal gland | 46 | 17.97 |  |  |  |
| Retroperitoneal region | 87 | 33.98 |  |  |  |
| Mediastinum | 90 | 35.16 |  |  |  |
| Other region | 25 | 9.77 |  |  |  |
| NA | 8 | 3.13 |  |  |  |
| ^a^ Two-sided *χ^2^* test for distributions between neuroblastoma patients and controls | | | | | |
